# Cognitive Behavioural Therapy Group Counselling for Nicotine Dependence in College Students: Behavioural and Resting‐State EEG Microstate Evidence

**DOI:** 10.1111/adb.70158

**Published:** 2026-04-21

**Authors:** Jiayue Zou, Can Zhang, Xiaoyu Dai, Shaoyu Tu, Haichao Zhao, Jiali Liu, Ofir Turel, Qinghua He

**Affiliations:** ^1^ Faculty of Psychology, MOE Key Laboratory of Cognition and Personality Southwest University Chongqing China; ^2^ Sichuan Electronic and Mechanic Vocational College Mianyang Sichuan China; ^3^ School of Computing and Information Systems The University of Melbourne Melbourne Australia

**Keywords:** cognitive behavioural therapy group counselling, nicotine dependence, resting‐state EEG microstate

## Abstract

Nicotine dependence is a chronic and relapsing disorder, with elevated onset risk during college years. Hence, we focus on college students and examine the immediate and short‐term effects of 6‐week cognitive behavioural therapy (CBT) group counselling in reducing nicotine dependence. Forty‐five participants were randomly assigned to either the CBT group (*n* = 24) or control group (*n* = 21) and completed the entire 6‐week intervention. Assessments included daily cigarette consumption, the Fagerström Test for Nicotine Dependence (FTND), DSM‐5 Criteria for Tobacco Use Disorder (DSM‐5), the Brief Questionnaire of Smoking Urges (QSU‐Brief) and a 5‐min eyes‐closed resting‐state EEG recording, administered at pre‐ and post‐intervention. Four weeks after intervention, FTND and QSU‐Brief were reassessed. Smokers in the CBT group demonstrated significant reductions in FTND, QSU‐Brief, DSM‐5 scores and daily cigarette consumption compared with the control group following the intervention. Moreover, QSU‐Brief scores in the CBT group remained lower than those in the control group at 4‐week follow‐up. Furthermore, greater reductions in daily cigarette consumption were accompanied by smaller decreases in microstate class B occurrence and coverage in the CBT group. These findings suggest that CBT group counselling could be an effective intervention for nicotine dependence.

## Graphical abstract

CBT group counselling effectively reduced nicotine dependence symptoms in both the immediate and short term, as also reflected in EEG microstate class B changes.

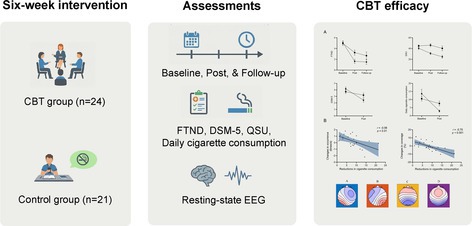

## 1 Introduction

Exposure to nicotine in young adulthood increases vulnerability to physical diseases, such as lung cancer [1], and psychiatric disorders [2, 3, 4, 5]. In addition to these health risks, nicotine dependence is characterized by extremely high relapse rates, reaching up to 90% among smokers who began smoking during young adulthood [2, 6, 7, 8]. Nevertheless, despite the severe and chronic consequences of nicotine dependence in young adulthood [9], smoking cessation among college students has received relatively little empirical attention [10]. Given that young adulthood is both the typical age of smoking onset and a pivotal stage for shaping long‐term health trajectories [11], developing targeted cessation strategies within university settings is essential to address this research gap and reduce both psychiatric and physical health risks associated with continued smoking.

Nicotine cessation has been approached through a variety of methods, including psychological and behavioural interventions, pharmacological treatments and mobile web‐based applications [12]. However, pharmacological approaches often face challenges such as potential side effects and low adherence among young adults, whereas mobile web‐based applications may lack the deep interpersonal support necessary for sustained abstinence. To address these limitations, cognitive behavioural therapy (CBT) offers a compelling alternative. Based on the core assumption that an individual's interpretation of reality shapes emotional and behavioural responses [13], CBT emphasizes the interaction among context, cognition, emotion and behaviour to help dismantle the cognitive distortions underlying nicotine dependence. Importantly, CBT‐based group counselling has been shown to be an effective nicotine cessation intervention [14, 15]. Furthermore, young adulthood is a period heavily influenced by peer dynamics and social environments. CBT‐based group intervention not only provides cognitive behavioural restructuring but also fosters a critical peer support network, utilizing positive peer influence to counteract withdrawal challenges. However, existing findings are limited, and few studies have examined its applicability and efficacy in college students. Furthermore, despite the clinical efficacy of CBT, the neurophysiological mechanisms underlying this clinical improvement remain largely unexplored. To bridge these gaps, it is crucial to simultaneously evaluate both the behavioural efficacy of CBT group counselling in college students and the neural mechanisms underlying its impact.

To address the neural mechanisms, we turn to EEG microstate analysis. It has been recognized as an effective method for investigating the neural characteristics underlying both fundamental cognitive processes, such as visual processing [16] and perceptual awareness [17], and neuropsychiatric disorders, with a particular focus on addictive behaviours including Internet and nicotine addiction [18, 19, 20]. Microstates are defined as brief, quasi‐stable patterns (40–120 ms) of brain activity, each corresponding to a distinct spatial configuration that recurs over time, and can be grouped into a specific class [21, 22, 23]. They are typically captured with three core parameters: microstate duration (average duration of a given class), occurrence (frequency of occurrence per second) and coverage (percentage of total duration within the overall analysis period) [24]. Different microstate classes have been linked to specific cognitive processes [21, 25]. Importantly, previous studies indicate that EEG microstates are sensitive to both the baseline severity of nicotine dependence and the acute reactivity to nicotine cues. On one hand, reflecting chronic dependence severity, the duration of microstate B is negatively correlated with the severity of cigarette consumption, showing distinct patterns compared to non‐smokers [19]. On the other hand, during state‐dependent cue reactivity, smoking cues elicit a significant increase in the duration of microstate classes B, C and D, with Class D duration serving as a potential biomarker for years of smoking [26]. Collectively, these findings highlight the utility of microstate parameters in capturing the multidimensional clinical features of nicotine dependence. Despite these advances, limited research has explored how microstate analysis can be applied to evaluate the effectiveness of CBT group counselling for nicotine dependence.

All in all, this study seeks to examine both immediate and short‐term effects of CBT group counselling on nicotine dependence among college students and to identify EEG microstate markers that may reflect neural changes associated with this intervention. We hypothesize that the CBT group will experience significant improvements in core symptoms of nicotine dependence, as also reflected in microstate class B changes following the intervention compared with the baseline.

## 2 Methods

### 2.1 Participants

Sample size was determined by an a priori power analysis using G*Power 3.1, indicating that a minimum of 34 participants would provide adequate power to detect the interaction effect between group and time. Fifty participants were recruited through online and campus flyers and randomly assigned to either CBT or control groups. All participants met the following inclusion criteria [27, 28]: (1) smoking at least one cigarette per day for 6 consecutive months prior to enrolment; (2) either scoring 3 or above on the Fagerström Test for Nicotine Dependence (FTND) [29] or meeting at least two symptoms from the DSM‐5 Criteria for Tobacco Use Disorder (DSM‐5) criteria for nicotine dependence [30]; (3) no history of substance addiction other than nicotine and no prior engagement in smoking cessation treatment; and (4) in good mental health, without anxiety and depression.

Five participants withdrew from the experiment due to personal reasons, and in the end, a total of 45 participants completed the entire 6‐week intervention: 24 in the CBT group (18 males, 6 females) and 21 in the control group (19 males, 2 females).

### 2.2 Experimental Design and Procedure

**FIGURE 1:**
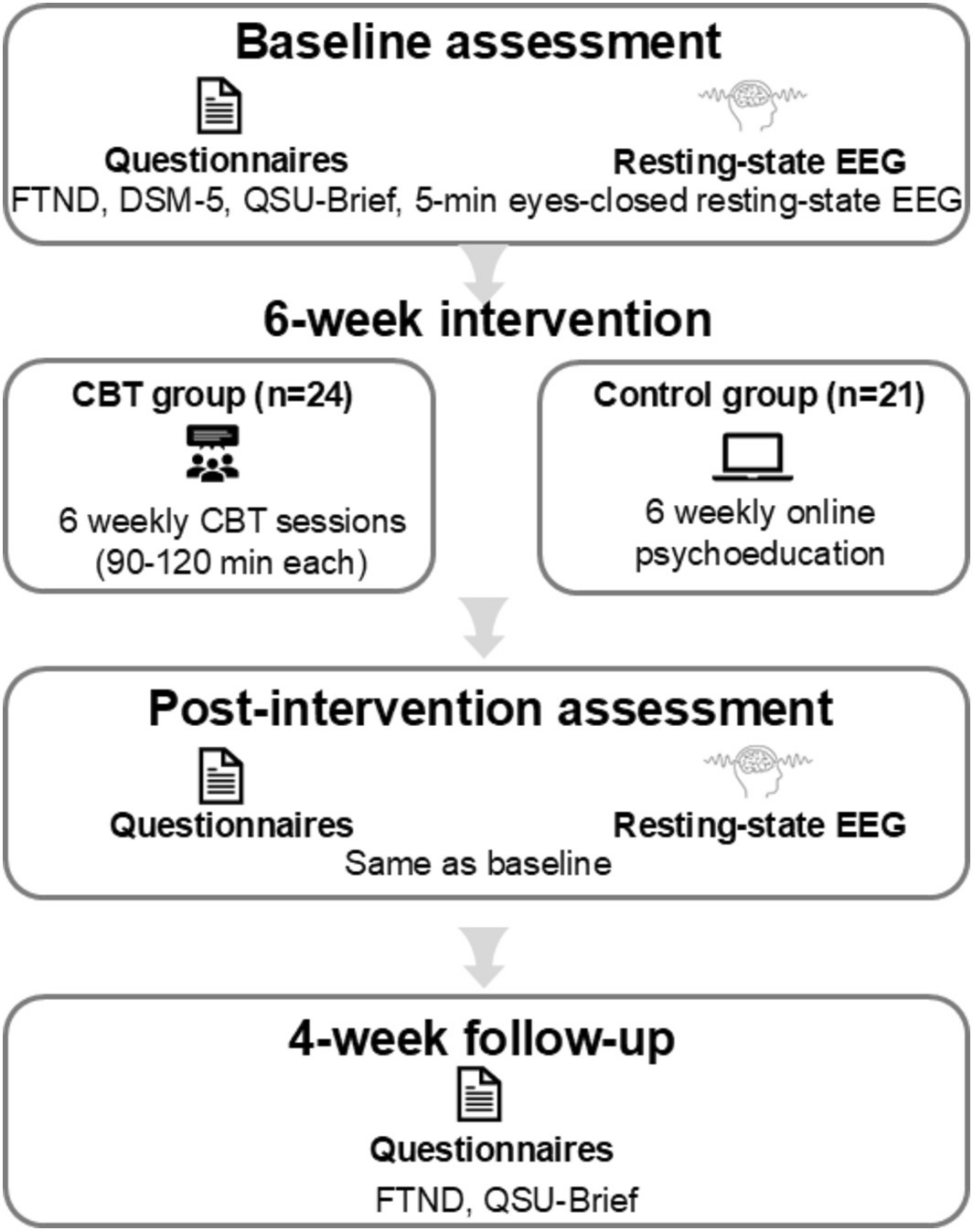
Experiment procedure.

The experiment (see procedure in Figure 1) employed a two‐factor mixed experimental design of 2 (Group: CBT group, control group) × 3 (Time: baseline, post‐intervention, 4‐week follow‐up). Prior to the intervention, all participants were instructed to complete self‐reported questionnaires, including the FTND, DSM‐5 and the Brief Questionnaire of Smoking Urges (QSU‐Brief). Following the questionnaires, a 5‐min resting‐state EEG with eyes closed was recorded. During the intervention, participants in the CBT group attended weekly CBT sessions for 6 consecutive weeks, whereas participants in the control group received six weekly online psychoeducation sessions. The control group intervention specifically involved posting information regarding healthy lifestyle practices and smoking‐related health risks weekly in an online chat group, which all participants completed. All intervention sessions were supervised by an experienced specialist, and each group was led by one professionally trained leader and one assistant. The post‐intervention assessment was identical to the baseline assessment. By comparing changes in questionnaire scores, we examined the immediate effects of CBT on nicotine dependence. Furthermore, we explored the neural biomarkers underlying the CBT effects by microstate EEG analysis. In addition, questionnaire data (FTND and QSU‐Brief) were collected again at the 4‐week follow‐up to examine the short‐term effects of CBT.

### 2.3 Instruments

#### 2.3.1 FTND

The FTND is a widely used standardized instrument for assessing nicotine dependence among smokers [29]. It consists of six items covering aspects such as the number of cigarettes smoked, the time of smoking and smoking situations. Items are differentially weighted, yielding a total score from 0 to 10. Higher scores indicate more severe nicotine dependence. Scores of 0–3 indicate low dependence, 4–6 indicate moderate dependence, and 7–10 indicate high dependence.

#### 2.3.2 DSM‐5

DSM‐5 diagnostic criteria are used to evaluate the presence and severity of nicotine dependence [30]. The criteria are assessed dichotomously (present/absent). In general, meeting two or more criteria within the past year is considered indicative of tobacco addiction.

#### 2.3.3 QSU‐Brief

The QSU‐Brief assesses subjective craving for smoking [31]. It includes 10 items rated on a 7‐point Likert scale (1 = *strongly disagree*, 7 = *strongly agree*). The Cronbach's α coefficients in the present study were 0.91 and 0.93.

#### 2.3.4 The Kessler Psychological Distress Scale (K10)

The Kessler Psychological Distress Scale (K10) is a brief measure of psychological distress [32]. It comprises 10 items assessing emotional states, each rated on a 5‐point scale from 1 (*none of the time*) to 5 (*all of the time*). Higher scores indicate higher psychological distress. The Cronbach's α coefficient of this scale in the present study was 0.93.

#### 2.3.5 Beck Depression Inventory‐II (BDI‐II)

The Beck Depression Inventory‐II (BDI‐II) is a widely used measure of depression severity [33]. It contains 21 items rated from 0 to 3, with higher total scores reflecting more severe depressive symptoms. The Cronbach's α coefficient of this scale in the present study was 0.92.

### 2.4 EEG Recording and Preprocessing

We acquired EEG using a 32‐channel NuAmps system (Compumedics Neuroscan) with Ag/AgCl electrodes positioned according to the international 10–20 system. AFz was used as the ground electrode. Electrodes were placed at the left (A1) and right (A2) mastoids, with the right mastoid serving as the online reference. Electrode impedances were kept below 5 kΩ. Data were digitized at 1000 Hz and subjected to a 400‐Hz low‐pass hardware filter during recording; no online high‐pass filtering was applied.

Offline processing was conducted in MATLAB R2019a with EEGLAB [34]. The pipeline involved band‐pass filtering between 1 and 40 Hz, resampling the data to 500 Hz, re‐referencing to the average reference, segmenting into 2‐s epochs, identifying and excluding ocular/muscle/body movement‐related components via ICA and discarding epochs exceeding ±100 μV.

### 2.5 Microstate Analysis

**FIGURE 2:**
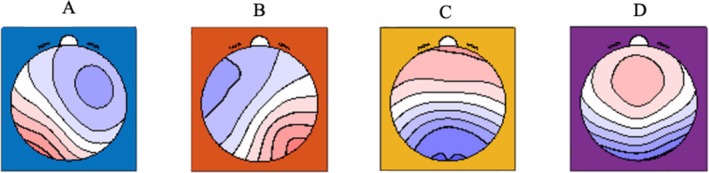
Microstate EEG topography of all participants.

Microstate analysis was conducted using the MICROSTATELAB toolbox in EEGLAB [24]. The K‐means clustering algorithm was applied to follow a standard procedure. (1) Individual microstate maps were first identified by clustering the scalp topographies corresponding to the peaks of the global field power (GFP) within each preprocessed EEG recording. GFP, defined as the standard deviation of all electrode potentials at a given time point, reflects the strength of the scalp electric field. (2) For each participant, the scalp voltage maps at GFP peak moments were submitted to K‐means clustering with 100 repetitions, specifying cluster numbers between 4 and 7, while ignoring map polarity during clustering. The optimal solution was determined as four clusters (see Figure 2). (3) These group‐level four microstate maps were then used as templates to back‐fit the continuous EEG data of each participant, resulting in a subject‐specific sequence of microstate A, B, C and D. (4) Finally, temporal parameters associated with each microstate class were extracted at the individual level. Specifically, the mean duration, frequency of occurrence and percent time covered were calculated [24, 35]. In our data, the global explain variance was 99.04%, with the CBT group contributing 77.20% and the control group contributing 76.97%.

### 2.6 CBT Group Counselling

In order to cater to the need of smoking cessation among college students, we designed group counselling programmes based on the principle of CBT [13] and smoking cessation guidelines [36]. The CBT group counselling consisted of six weekly, 90‐ to 120‐min sessions, each covering a specific theme: orientation and goal setting, identification of irrational beliefs, integrating motivation with action, reconstruction training for smoking‐related behaviours, relapse prevention and consolidation and future planning (see Table S1 for details).

### 2.7 Statistical Analysis

All behavioural data were analysed using ANOVA in SPSS 23.0 to compare intervention effects between the two groups. Building on prior evidence that microstate class B may serve as a biomarker of nicotine dependence severity [19], we examined changes from baseline to post‐intervention in temporal parameters of Class B within the CBT group to identify biomarkers of CBT‐related smoking reduction by using paired‐sample *t*‐tests. We further explored the relationship between behavioural and microstate measures using Spearman correlation.

## 3 Results

### 3.1 Baseline Characteristics

The CBT group and control group were matched on demographic characteristics, mental health (K10 and BDI‐II; see scale names in the notes for Table 1) and the levels of nicotine dependence (smoking duration, daily cigarette consumption, FTND, DSM‐5 and QSU‐Brief scores). The detailed statistics can be found in Table 1.

**TABLE 1:** Baseline characteristics and group comparisons.

Variable	CBT group (*N* = 24)	Control group (*N* = 21)	Test statistic	*p*
Sex (male/female)	18/6	19/2	χ^2^(1) = 1.84	0.18
Age (year)	20.45 ± 1.86	20.57 ± 1.80	*t*(43) = 0.29	0.77
Smoking duration (year)	3.83 ± 2.24	3.43 ± 1.94	*t*(43) = 0.64	0.52
Daily cigarette consumption	13.63 ± 13.19	10.48 ± 6.29	*t*(43) = 0.10	0.32
FTND	5.08 ± 1.61	4.95 ± 1.80	*t*(43) = 0.26	0.80
DSM‐5	4.17 ± 1.31	3.71 ± 1.27	*t*(43) = 1.17	0.25
QSU‐Brief	40.54 ± 12.57	44.86 ± 8.49	*t*(43) = 1.33	0.19
K10	29.71 ± 8.88	25.62 ± 9.35	*t*(43) = 1.50	0.14
BDI‐II	13.88 ± 8.99	12.14 ± 12.99	*t*(43) = 0.53	0.60

Abbreviations: BDI‐II, Beck Depression Inventory‐II; DSM‐5, Diagnostic and Statistical Manual of Mental Disorders, Fifth Edition (criteria for tobacco use disorder); FTND, Fagerström Test for Nicotine Dependence; K10, Kessler Psychological Distress Scale; QSU‐Brief, Brief Questionnaire of Smoking Urges.

### 3.2 Behavioural Results for Immediate CBT Effects

**FIGURE 3:**
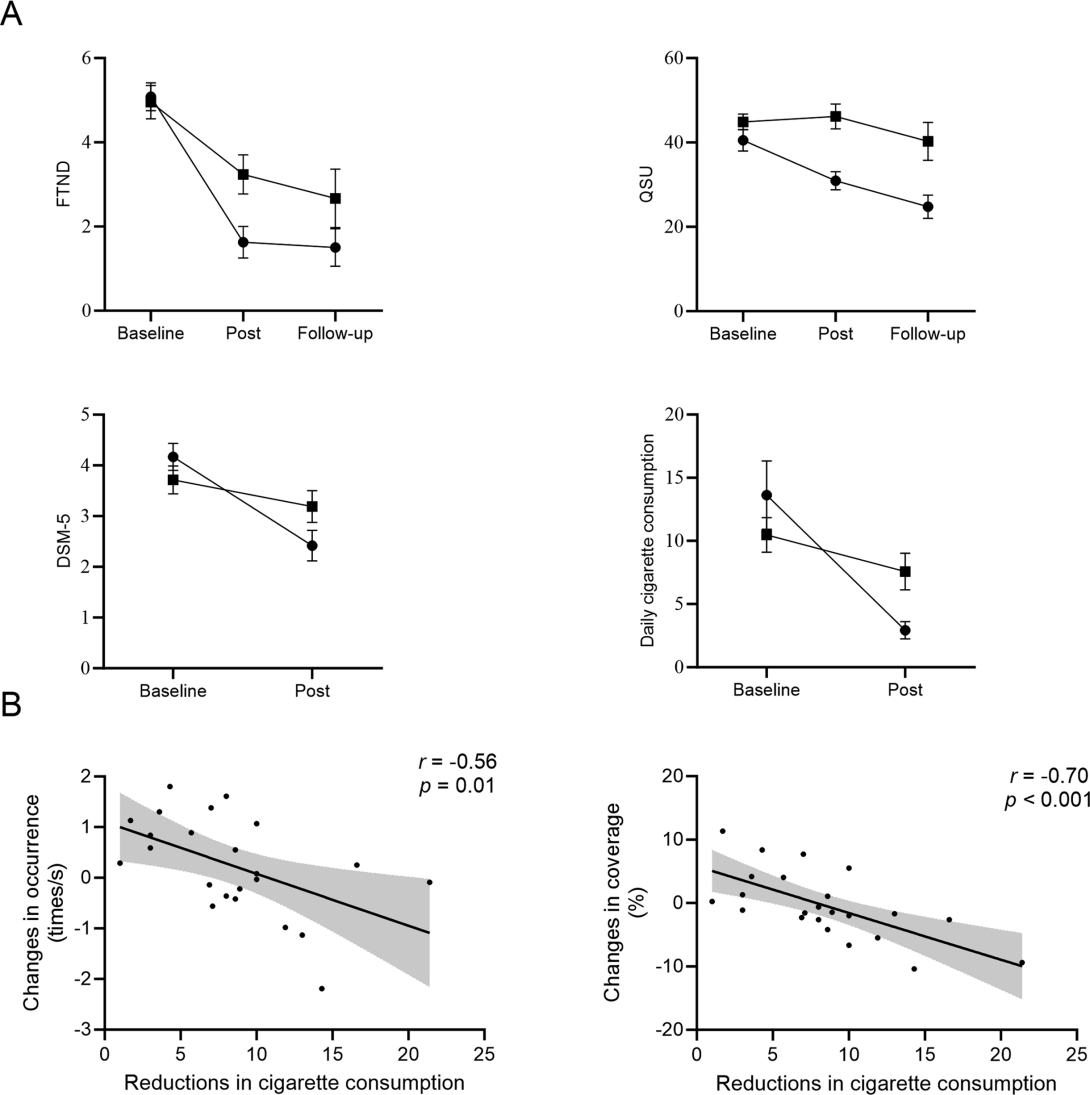
(A) Changes in behavioural results across time in the CBT and control groups; Error bars represent the standard error of the meant (SEM). (B) Correlations between reduction in cigarette consumption and changes in occurrence and coverage of class.

A 2 (Group: CBT vs. Control) × 2 (Time: baseline vs. post‐intervention) repeated‐measures ANOVA revealed significant Group × Time interaction effects for FTND, QSU‐Brief, DSM‐5 and number of cigarettes consumed per day (Table 2). Participants in the CBT group showed significant reductions from baseline to post‐intervention in FTND, QSU‐Brief, DSM‐5 and daily cigarette consumption, whereas no significant changes were observed in the control group (Figure 3A).

**TABLE 2:** Behavioural results by group and time.

Variables	CBT group (*N* = 24, *M* ± SD)		Control group (*N* = 21, *M* ± SD)		Group × Time *F*_1,43_	*p*	$\eta_{p}^{2}$
	Baseline	Post	Baseline	Post			
FTND	5.08 ± 1.61	1.63 ± 1.84	4.95 ± 1.80	3.24 ± 2.14	4.33	0.04	0.09
QSU‐Brief	40.54 ± 12.57	30.92 ± 10.67	44.86 ± 8.49	46.19 ± 13.62	5.37	0.03	0.11
DSM‐5	4.17 ± 1.31	2.42 ± 1.47	3.71 ± 1.27	3.19 ± 1.44	7.12	0.01	0.14
Daily cigarette consumption	13.63 ± 13.19	2.92 ± 3.34	10.48 ± 6.29	7.57 ± 6.69	7.83	0.01	0.15

In addition, there were significant main effects of Time for FTND (*F*_1,43_ = 38.10, *p* < 0.001, $\eta_{p}^{2}$ = 0.47), daily cigarette consumption (*F*_1,43_ = 23.87, *p* < 0.001, $\eta_{p}^{2}$ = 0.36) and DSM‐5 (*F*_1,43_ = 24.47, *p* < 0.001, $\eta_{p}^{2}$ = 0.36), reflecting overall reductions from baseline to post‐intervention. Significant main effects of Group were observed for FTND (*F*_1,43_ = 4.23, *p* = 0.046, $\eta_{p}^{2}$ = 0.09) and QSU‐Brief (*F*_1,43_ = 15.36, *p* < 0.001, $\eta_{p}^{2}$ = 0.26), indicating lower FTND and QSU‐Brief scores in the CBT group compared with the control group.

### 3.3 Behavioural Results for Short‐Term CBT Effects

Of the original sample, 37 participants completed the follow‐up assessment (CBT group: *n* = 22; Control group: *n* = 15), yielding an 82.22% follow‐up rate. A 2 (Group: CBT group, control group) × 3 (Time: baseline, post‐intervention, follow‐up) repeated‐measures ANOVA revealed no significant interaction effects for either QSU‐Brief or FTND (*p*s > 0.05). However, independent‐samples *t*‐tests at follow‐up indicated that QSU‐Brief scores were significantly lower in the CBT group (24.77 ± 13.01) than in the control group (40.27 ± 17.44, *t*(35)= 3.10, *p* = 0.004, Cohen's *d* = 1.04). FTND scores did not differ between groups at follow‐up (*p* > 0.05).

### 3.4 Microstate Class B Results Within CBT Group

Paired‐sample *t*‐tests showed no significant differences in duration, occurrence or coverage of Class B from baseline to post‐intervention within CBT group (*p*s > 0.05; see Table S2). We next examined the relationships between reductions in nicotine dependence scores and changes in Class B temporal parameters. One participant in the CBT group showed an extreme reduction in cigarette consumption (> 3 SD) and was excluded from analyses. The results revealed that the larger reductions in cigarette consumption were associated with smaller decreases in occurrence (*r* = −0.56, *p* = 0.01) and coverage (*r* = −0.70, *p* < 0.001) of Class B (see Figure 3B). Notably, inclusion of the outlier did not change the direction or significance of these associations. No other significant correlations were found between the temporal parameters of microstate class B and the FTND, QSU‐Brief or DSM‐5 scores (Table S3).

## 4 Discussion

The present study sought to examine the efficacy of CBT group counselling for nicotine dependence among college students, with a particular focus on both smoking‐related behavioural outcomes and EEG microstate alterations. Consistent with our hypothesis, smokers in the CBT group displayed significant reductions in various smoking dependence metrics (FTND, QSU‐Brief and DSM‐5 scores and daily cigarette consumption) after 6‐week intervention, compared with the control group. Importantly, QSU‐Brief scores in the CBT group remained lower than those in the control group at the 4‐week follow‐up, suggesting that CBT group counselling may produce sustained effects on craving reduction. Furthermore, despite the lack of significant pre–post changes in the temporal parameters of microstate class B at the group level, our results revealed an association between behavioural and neural indicators at the individual level: greater reductions in daily cigarette consumption were accompanied by smaller decreases in microstate class B occurrence and coverage. These findings highlight not only the clinical effectiveness of CBT group counselling in reducing nicotine addiction symptoms among college students but also its potential to modulate underlying neural processes.

Following a 6‐week intervention, the CBT group displayed reductions in scores on a self‐report measure of nicotine dependence, whereas the control group showed no changes. In addition, the scores of smoking craving in the CBT group still remained lower than those in the control group at follow‐up. These findings are in line with previous research, suggesting that CBT is effective in reducing nicotine dependence [14, 37]. We developed a group counselling programme grounded in CBT principles aimed at reducing nicotine dependence and promoting smoking cessation. The intervention was mainly structured into two major components: The cognitive component aimed to modify maladaptive attitudes and beliefs related to smoking, whereas the behavioural component focused on promoting the substitution of smoking‐related behaviours with healthier alternatives [36]. These techniques can be effectively applied across different stages of managing smoking behaviour, including preparation, quit attempt and relapse prevention.

Decreases in occurrence and coverage of microstate class B were negatively correlated with reductions in daily cigarette consumption. This finding aligns with previous evidence suggesting that microstate class B serves as a neural marker of nicotine dependence [19]. Previous EEG‐fMRI studies have suggested a close association between EEG microstates and fMRI‐defined resting‐state networks (RSNs) [25, 38, 39]. Specifically, microstate class B has been linked to the visual RSN [38]. Because addicted individuals often exhibit biased visual processing towards addiction‐related stimuli, microstate B may be particularly associated with visual and addiction‐related cue processing. For example, studies on methamphetamine‐dependent patients reported significantly higher coverage and occurrence of class B during the cue‐induced condition compared to the no‐cue resting state [20]. In addition to the influence of addiction‐related visual cues, addiction itself may impair microstate class B. For instance, a study found that the duration of microstate B was shorter in smokers than in non‐smoking controls and negatively correlated with years of smoking [19]. Similarly, other studies have shown that methamphetamine‐dependent patients exhibited lower temporal parameters (duration, occurrence and coverage) of Class B compared with healthy controls [18, 20, 40]. All in all, supplementing previous findings, the present study demonstrates that a larger reduction in cigarette consumption was associated with smaller decreases in the occurrence and coverage of microstate class B, thereby supporting the effectiveness of CBT group counselling for smokers.

Several limitations of our study should be acknowledged. First, the sample size was relatively small, which constrained the generalizability of the results. Expanding the sample size of smokers in future studies would enhance the credibility and applicability of the findings. Second, the follow‐up period was limited to 4 weeks after the intervention, and we did not collect resting‐state EEG at follow‐up. As a result, the long‐term effects of CBT group counselling on nicotine dependence and the underlying neural changes could not be fully examined. Finally, regarding the neurophysiological evaluation, our study focused solely on microstate analysis to evaluate the effectiveness of CBT group counselling. To achieve a more comprehensive understanding of the neural mechanisms underlying nicotine dependence, task‐based EEG [27] and multimodal fMRI [28, 41] should also be considered in future research.

## 5 Conclusion

This study examined both the immediate and short‐term effects of CBT group counselling on nicotine dependence among college students. The findings demonstrated that CBT group counselling effectively reduced nicotine dependence symptoms in both immediate and short term, as also reflected in microstate class B changes. This means that microstate class B may serve as a potential neural marker for evaluating the efficacy of CBT group counselling in reducing nicotine dependence.

## Author Contributions

Jiayue Zou, Can Zhang and Qinghua He designed the study. Jiayue Zou, Can Zhang, Xiaoyu Dai and Shaoyu Tu carried out recruitment of subjects and collection of their behavioural and EEG data. Jiayue Zou and Can Zhang conducted the data analysis. Jiayue Zou drafted the manuscript. Qinghua He, Ofir Turel, Haichao Zhao and Jiali Liu provided critical revisions of the manuscript.

## Funding

This work was supported by research grants from the National Natural Science Foundation of China (31972906) and the Graduate Research Innovation Project of Southwest University (SWUS24034).

## Conflicts of Interest

The authors declare no conflicts of interest.

## Supporting information


**Table S1:** CBT group counselling programmes.
**Table S2:** Microstate class B results within CBT group (*n* = 24).
**Table S3:** Correlations between temporal parameters of EEG microstate class B and measures of nicotine dependence.

## Data Availability

Researchers who require access may contact us via email, and we will provide the corresponding data and code based on the specific requests.
