# Supplementary material for: Cognitive Behavioural Therapy Group Counselling for Nicotine Dependence in College Students: Behavioural and Resting‐State EEG Microstate Evidence
Source: Addict Biol. 2026 Apr 21;31(4):e70158. doi: 10.1111/adb.70158 (PMC13100488; doi:10.1111/adb.70158)
Supplement: Supplementary file 1 — Table S1: CBT group counselling programmes. Table S2: Microstate class B results within CBT group (n = 24). Table S3: Correlations between temporal parameters of EEG microstate class B and measures of nicotine dependence. [file ADB-31-e70158-s001.docx]

**SUPPLEMENTARY MATERIALS**

**Table S1.** CBT group counseling programmes

| Theme | Objective | Activities and Processes |
| --- | --- | --- |
| Orientation and Goal Setting | To establish a foundation for effective group participation through developing shared understanding, formulating a group contract, enhancing program motivation, and clarifying individual expectations and goals. | 1. Introduction and warm up: presentation of the group counseling program goals and structure, followed by warm up activities (Ice-breaking activities); establishment of a group contract. 2. Quit-Day setting. 3. Formulation of individual cessation plans. 4. Smoking cessation skills training. 5. Homework: Smoking dairy |
| Identification of Irrational Beliefs | To help participants identify negative automatic thoughts in daily life and recognize irrational patterns of thinking. | 1. Review and feedback on last session. 2. Cognitive analysis of smoking-related thoughts. 3. Homework: Daily thought record. |
| Integrating Motivation with Action | To transform reasons for quitting smoking into practical behaviors by applying a circular “situation-cognition-behavior-result” model. Participants accumulate successful experiences through small goals, enhance self-efficacy, and strengthen group support by publicly committing to reward and punishment rules, thereby reducing the likelihood of relapse. | 1. Review and feedback 2. Establishment of reward and punishment mechanisms 3. Training in managing withdrawal symptoms 4. Homework: Applying model in next week. |
| Reconstruction Training for Smoking-Related Behaviors | To weaken physiological and psychological connections to nicotine dependence through behavioral substitution strategies and modification of smoking-associated daily routines. | 1. Review and feedback. 2. Adjustment of individual cessation plan. 3. Modification of daily habits related to smoking. 4. Homework: Practice substitution strategies. |
| Relapse Prevention | To prevent relapse by addressing sudden cravings and challenging rationalization tendencies that may justify smoking. | 1. Review and feedback. 2. Adjustment of individual cessation plan. 3. “Challenging the rationalization” exercise. 4. Homework: Trigger log. |
| Consolidation and Relapse Prevention | To strengthen participants’ self-awareness of smoking-triggers, enhance generalization of coping strategies, and establish long-term smoking cessation plans. | 1. Review and feedback 2. Sharing of overall changes achieved during the five prior sessions. 3. Development of long-term cessation plans. 4. Completion of a satisfaction survey. |

**Table S2.** Microstate class B results within CBT group (*n* = 24)

| Parameter | Baseline | Post | *t* (23) | *p* |
| --- | --- | --- | --- | --- |
| Duration | 0.038 ± 0.001 | 0.041 ± 0.001 | -1.77 | 0.09 |
| Occurrence | 5.50 ± 1.17 | 5.23 ± 1.03 | 1.37 | 0.38 |
| Coverage (%) | 20.97 ± 4.86 | 21.35± 4.85 | -0.35 | 0.73 |

**Table S3.** Correlations between temporal parameters of EEG microstate class B and measures of nicotine dependence

| Parameter | Δ Occurrence | Δ Coverage | Δ Duration |
| --- | --- | --- | --- |
| Δ FTND | *r* =0.35  *p* =0.10 | *r* =0.09  *p* =0.69 | *r* = -0.19  *p* = 0.38 |
| Δ QSU-Brief | *r* =0.02  *p* =0.91 | *r* = -0.17  *p* =0.45 | *r* = -0.39  *p* = 0.06 |
| Δ DSM-5 | *r* =-0.25  *p* =0.26 | *r* =-0.09  *p* =0.69 | *r* =0.02  *p* =0.94 |

Note. *r*: Spearman correlation coefficient. Δ represents the change in scores or parameters from pre- to post-intervention. FTND: the Fagerström Test for Nicotine Dependence; QSU-Brief: the Brief Questionnaire of Smoking Urges; DSM-5 = Diagnostic and Statistical Manual of Mental Disorders, Fifth Edition (criteria for tobacco use disorder).
